# Spontaneous serum autoantibody fluctuations: *To be or not to be*

**DOI:** 10.1038/s41380-020-00883-4

**Published:** 2020-09-23

**Authors:** Hannelore Ehrenreich, Justus Wilke, Agnes A. Steixner-Kumar

**Affiliations:** Clinical Neuroscience, Max Planck Institute of Experimental Medicine, Göttingen, Germany

**Keywords:** Molecular biology, Biomarkers

With the phrase *“To be, or not to be – that is the question”*, William Shakespeare’s Hamlet considers in essence non-existence versus existence. In a figurative sense, this soliloquy comes to mind in connection with a still mysterious drama of nature, namely the seemingly unpredictable appearance or vanishing of circulating autoantibodies (AB). This unexplored phenomenon is not only of biological but likely of considerable clinical and diagnostic importance [1, 2]. At present, we do not understand the physiological significance of AB in general and of AB against N-methyl-D-aspartate-receptor subunit-NR1 (NMDAR1-AB) in particular. Their high frequency and presence across mammals, however, points against a purely “pathological significance” [3–6].

Initially assuming a pathological role of AB, Pollak et al. studied for the first time in serum samples of a cohort of 254 subjects at clinical high risk for psychosis (CHR) versus 116 healthy controls (HC) multiple neuronal antigens, implicated in CNS autoimmune disorders [2]. The authors’ main outcome of interest turned out negative: AB do not predict transition to psychosis, which is undoubtedly a clinically important message. Additionally, they made several unexpected preliminary observations worth pursuing. Since such CHR cohort is extremely difficult to recruit and follow-up, the number of individuals is respectable but, at the same time, it is not sufficient for firm conclusions on the pathophysiology of AB – a typical problem of small numbers, shared by most publications of peers in the field, which the authors themselves realize, and wisely observe caution not to over-interpret their findings. This is also why they primarily focus on NMDAR1-AB as the most frequently found AB, despite having explored AB to 32 different antigens [2].

NMDAR1-AB are frequent and can “easily” be checked for functionality – thus are convenient tools for study, as they likely stand for numerous other brain-directed AB exerting effects according to their epitopes and respective functionality [6, 7]. Hence, we have to be aware that we are just seeing the tip of the iceberg. A good example of our scratching on the surface is given by Pollak et al. [2]: The authors find binding to brain (hippocampus, cerebellum) of sera from seven “negative” subjects (CHR as well as healthy), implicating unknown AB in the circulation.

Another example for by chance findings based on small numbers is the lack of NMDAR1-AB of the IgA class in healthy controls [2], which is not seen in large samples [3, 5]. Similar problems apply for described associations with psychopathology, cognition, or global assessment of functioning which are all highly interesting first signals but rest on tiny numbers and require replication in large samples [2]. This extends to the results of fixed versus live cell-based assays (compared only for NMDAR1-AB of the IgG class) with the latter yielding higher numbers for still unknown reasons which possibly include higher sensitivity or unspecific cross-reactions in live cultures. Importantly, however, the results for CHR and HC were comparable also in live cell-based assays [2]. Also in this context, associations of the different assay results with clinical readouts are still highly preliminary. Importantly, the overall accessibility of AB to the brain in these cohorts has remained unclear. Information on an important proxy of accessibility, blood–brain–barrier (BBB) function, might have rendered one or the other finding clearer [8]. Serum S100B levels are problematic as the sole criterion, among others because of additional peripheral S100B expression [9]. Also the albumin quotient has its limitations indicating a disturbed blood–cerebrospinal fluid barrier, but at least for clinical routine delivers a solid estimation of BBB disturbance [10]. Nevertheless, even upon intact BBB, at least low amounts of circulating AB reach the CNS (“immunoprecipitator” role of the brain) and can potentially exert phenotypical effects [11, 12]. The location of AB transfer to the brain (e.g., circumventricular organs) – also in healthy conditions – may co-determine the potential functions of even low amounts of AB which perhaps just “mildly modulate” our behavior. Indeed, we hypothesize that AB modify behavior and brain functions in an “epigenetic-like fashion”. They are induced and boosted mainly by environmental stimuli (e.g., infections, chronic life stress, brain lesions [1, 5, 11]). Furthermore, genetic predisposition – at least for some AB – obviously plays a role [5].

Intriguingly, in the paper of Pollak et al., presence of any neuronal AB was associated with larger amygdala volumes. This fits perfectly with stress as inducer of both AB [1, 4] and amygdala enlargement upon chronic stress [13]. Indeed, we find accumulated seroprevalence of 23 brain-directed AB in young migrants versus nonmigrants increased, suggesting a global inducer role of chronic stress for humoral autoimmunity [1, 4]. The underlying inducing or boosting mechanisms, however, remain to be determined.

In this sense, also fluctuations of NMDAR1-AB may have to be understood [1, 4, 5]: Boosters disappear and titers fall. However, specific AB producing clones apparently stay on, mostly polyclonal, and remain temporarily silent. Later, boosters reappear and titers rise again. Pollak et al. nicely confirm these fluctuating titers [2]. Missing thus far is any information on the predictive value of AB fluctuations for fluctuating phenotypes. In the case of NMDAR1-AB, the AB act ketamine-like, display “effects and side effects” – some advantageous (e.g., antidepressive, stroke lesion-reducing [1, 14]), others unwanted (inducing psychosis or promoting dementia [12, 15–17]) – just as with pharmacological treatments. Following these effects in association with AB fluctuations will be important tasks for future studies.

Dependent on the circumstances, i.e., absence or presence of an underlying brain inflammation, NMDAR1-AB or other brain-directed AB in higher amounts may contribute to more chronic processes (dementia, psychosis, epilepsy) or fulminant courses (“anti-NMDAR encephalitis”), provided access to the brain upon BBB dysfunction or intrathecal synthesis by respective B cell clones in presence or even absence of specific T cells. In this context, the formation of ectopic lymphoid follicles in the meninges (as shown in patients with MS [18]) could represent a critical step in maintaining humoral autoimmunity and in modulating brain functions including behavior or in disease exacerbation.

In the case of NMDAR1-AB, we will have to start exploring as well effects on cells other than neurons as far as they express the antigen. This can for instance sophisticatedly be screened using available scRNA-seq data which may result in detecting NMDAR expressing subclusters. On top of transcriptional findings, protein expression and even more importantly, functionality will have to be demonstrated. NMDAR function in non-neuronal cells is not too well understood and it is unknown whether internalization similar to neuronal NMDAR takes place.
